# Domain wall motion driven by a wide range of current in coupled soft/hard ferromagnetic nanowires

**DOI:** 10.1039/d1na00540e

**Published:** 2022-02-03

**Authors:** Ziyang Yu, Bin Gong, Lun Xiong, Xinran Du, Chenhuinan Wei, Rui Xiong, Zhihong Lu, Yue Zhang

**Affiliations:** Hubei Key Laboratory of Optical Information and Pattern Recognition, School of Optical Information and Energy Engineering, Wuhan Institute of Technology Wuhan 430205 P. R. China xionglun@wit.edu.cn; Hubei Provincial Key Laboratory of Green Materials for Light Industry, Hubei University of Technology Wuhan 430068 China; Key Laboratory of Artificial Micro- and Nano-structures of Ministry of Education, School of Physics and Technology, Wuhan University Wuhan 430072 China; The State Key Laboratory of Refractories and Metallurgy, School of Materials and Metallurgy, Wuhan University of Science and Technology Wuhan 430081 China; School of Optical and Electronic Information, Huazhong University of Science and Technology Wuhan 430074 China yue-zhang@hust.edu.cn

## Abstract

Racetrack memory with the advantages of small size and high reading speed is proposed based on current-induced domain wall (DW) motion in a ferromagnetic (FM) nanowire. Walker breakdown that restricts the enhancement of DW velocity in a single FM nanowire can be depressed by inter-wire magnetostatic coupling in a double FM nanowire system. However, this magnetostatic coupling also limits the working current density in a small range. In the present work, based on micromagnetic calculation, we have found that when there is a moderate difference of magnetic anisotropy constant between two FM nanowires, the critical current density for triggering the DW motion can be reduced while that for breaking the inter-wire coupling can be enhanced significantly to a magnitude of 10^13^ A m^−2^, which is far above the working current density in current electronic devices. The manipulation of working current density is relevant to the modification of DW structure and inter-wire magnetostatic coupling due to the difference of the anisotropy constants between the two nanowires and paves a way to develop racetrack memory that can work in a wide range of current.

## Abstract 2

A double FM nanowire system composed of two FM nanowires with distinct magnetic anisotropy constants (the lower DW is induced to move under the injection of current, and the upper one is also dragged to move by the interlayer magnetostatic coupling).

## Graphical abstract

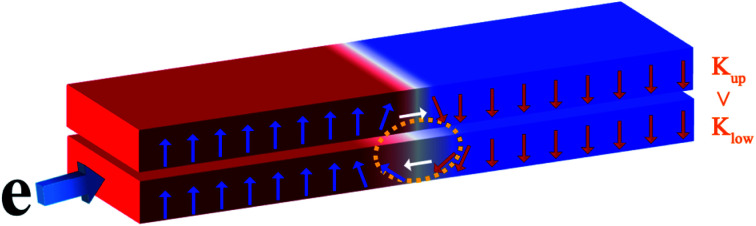

## Introduction

In racetrack memory that was proposed by Prof. Stuart S. P. Parkin of IBM in 2008,^1^ digital information is stored in an array of magnetic domains in a nanowire of ferromagnetic metal (FM), and the domain wall (DW) that separates the neighboring domains is able to be driven by injecting an electrical current due to the spin-transfer torque (STT) effect.^2–4^ When compared to a traditional hard disc driver, the racetrack memory device exhibits advantages of small size and high reading speed. Therefore, the current induced DW motion (CIDWM) in an FM nanowire has been widely investigated in experiments and in theory.^5–12^

In CIDWM, the stability of DW structure depends on current density. When the current density exceeds a critical value, the magnetic moments in the DW precess, and the DW motion slows down. This is the so-called Walker breakdown.^8,13–15^ Depression of the Walker breakdown results in DW motion at a high velocity with a stable DW structure. In 2015, Purnama *et al.* found that in an FM/insulator/FM sandwiched nanowire with perpendicular magnetic anisotropy (PMA), the magnetostatic coupling between the DWs in the neighboring FM nanowires provides a closed magnetic line of force that stabilizes the DW structure and depresses the Walker breakdown.^16^ The depression of Walker breakdown by inter-wire magnetostatic coupling has also been observed experimentally very recently.^17^ However, the inter-wire magnetostatic coupling also restricts the range of working current, and the DW can only be induced to move with the current density between two critical values. One is the smallest current density (*J*_a_) to overcome the pinning due to the inter-wire magnetostatic coupling.^16^ Lowering *J*_a_ reduces dissipation. On the other hand, when the current density is higher than another critical value (*J*_b_), the two DWs are decoupled. When the current density is between *J*_a_ and *J*_b_, the Walker breakdown is depressed.^16^ Therefore, a low *J*_a_ with a high *J*_b_ is expectable in such a double FM nanowire system. However, in a sandwiched multilayer composed of two FM nanowires with identical parameters, lowering *J*_a_ and enhancing *J*_b_ at the same time seems not easy. This is because the magnetostatic coupling acting on the two DWs is the same, and they become stronger or weaker together. As a result, the range of current density for the synchronization of DW motion is fixed in a small range.

In the present work, we have found that the small difference of the magnetic anisotropy constant between the two nanowires is able to reduce *J*_a_ and enhance *J*_b_ at the same time. As a result, the gap between *J*_a_ and *J*_b_ is significantly enlarged, and *J*_b_ becomes even higher than 10^13^ A m^−2^, which is far beyond the current density that can be injected in current electronic devices.

## Methods

**Fig. 1:**
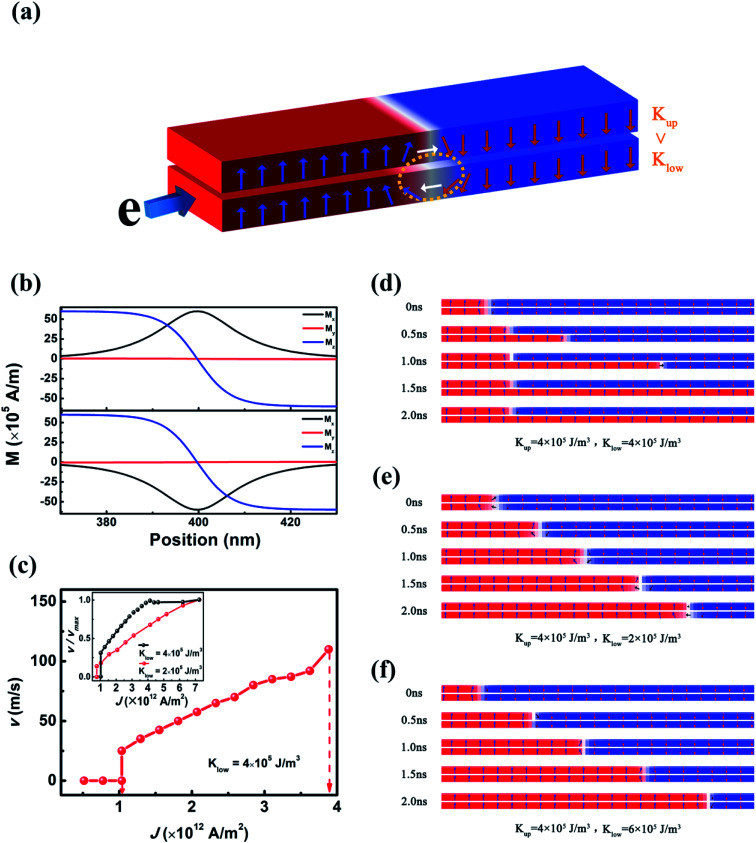
(a) Model: the double FM nanowire system composed of two FM nanowires with distinct magnetic anisotropy constants (the lower DW is induced to move under the injection of current, and the upper one is also dragged to move by the interlayer magnetostatic coupling); (b) *x*, *y*, and *z* components of magnetization near the centre of the DW in the two nanowires with *K*_l_ = *K*_u_ = 4 × 10^5^ J m^−3^; (c) average velocity for the lower DW in the double nanowire system with *K*_l_ = *K*_u_ = 4 × 10^5^ J m^−3^ as a function of current density (*J*) (inset: comparison of the normalized velocity for the lower DW in the double nanowire system with *K*_l_ = *K*_u_ = 4 × 10^5^ J m^−3^ and that with *K*_l_ = 2 × 10^5^ J m^−3^ and *K*_u_ = 4 × 10^5^ J m^−3^); (d–f) snapshots of DW motion under *J* = 7.5 × 10^12^ A m^−2^ for *K*_l_ = *K*_u_ = 4 × 10^5^ J m^−3^, *K*_l_ = 2 × 10^5^ J m^−3^ and *K*_u_ = 4 × 10^5^ J m^−3^, and *K*_l_ = 6 × 10^5^ J m^−3^ and *K*_u_ = 4 × 10^5^ J m^−3^.

The CIDWM is simulated using the micromagnetic simulation software named Object-Oriented Micromagnetic Framework (OOMMF). In the simulation, we consider the CIDWM in a double FM nanowire system with PMA, and the magnetic anisotropy constants of the upper (*K*_u_) and lower (*K*_l_) nanowires are different (Fig. 1(a)). The interfacial Dzyaloshinskii–Moriya interaction (DMI) is also taken into account since it usually exists in an ultrathin FM film with PMA and contributes to stabilizing the Néel-type DW structure.^17,18^ The current is injected into the lower nanowire, in which the DW is driven due to the STT effect. Because of the inter-layer magnetostatic coupling, the DW in the upper one is also dragged to move to reduce the demagnetization energy till the current density is above *J*_b_.

The simulation is based on resolving the Landau–Lifshitz–Gilbert (LLG) equation containing the STT terms:^8^1
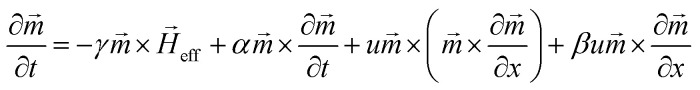


In eqn (1), the parameters 
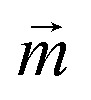
, *t*, and *γ* represent the unit vector for the orientation of magnetic moment, time, and the absolute value of gyromagnetic ratio. The first and second terms at the right side of eqn (1) contribute to the torque from the effective magnetic field 
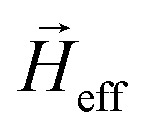
 and Gilbert damping with the damping coefficient *α* = 0.01, respectively. The third and fourth terms are the adiabatic and non-adiabatic STT with the coefficient *β* = 0.04,^16^ respectively. Here *β* is significantly larger than *α*, which will lead to the STT-induced Walker breakdown under a small *J*.^8^*u* is the velocity of an electron: 
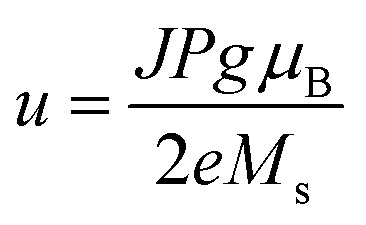
, where *J* is the current density, *P* is the polarization rate (*P* = 0.7), *g* is the Landé factor, *μ*_B_ is the Bohr magneton, and *e* is the electron charge.

The model and parameters are as follows: the length and width of the nanowire are 5000 nm and 40 nm, respectively. The lower nanowire and the upper one share the same thickness (6 nm), and the inter-wire distance is 2 nm. The cell dimensions are 5 nm × 5 nm × 2 nm. The saturation magnetization (*M*_S_) is 6 × 10^5^ A m^−1^. The exchange stiffness constant (*A*) is 1.3 × 10^−11^ J m^−1^. The absolute value of the DMI constant (*D*) is between 0 mJ m^−2^ and 1 mJ m^−2^, and the signs of *D* of the two nanowires are opposite. The *K*_u_ is fixed as 4 × 10^5^ J m^−3^, and the *K*_l_ changes between 2 × 10^5^ J m^−3^ and 6 × 10^5^ J m^−3^.

## Results and discussion

Initially, the Néel-type DWs are generated in the two nanowires (Fig. 1(b)). Because of their opposite chirality, a closed magnetic line of force can be generated between the two nanowires.^16,17^ When both *K*_u_ and *K*_l_ are 4 × 10^5^ J m^−3^, *J*_a_ and *J*_b_ are 0.9 × 10^12^ A m^−2^ and 3.7 × 10^12^ A m^−2^, respectively (Fig. 1(c)). When *J* is 7.5 × 10^12^ A m^−2^, higher than this *J*_b_, the lower DW is able to be driven by STT, but the upper DW is decoupled with the lower one (Fig. 1(d)). However, when *K*_l_ is 2 × 10^5^ J m^−3^ or 6 × 10^5^ J m^−3^, the upper DW can still be dragged to move together with the lower one under the same *J* (Fig. 1(e) and (f)). This indicates that the small difference of the magnetic anisotropy constant between the two nanowires contributes to the enhancement of *J*_b_. Additionally, when *K*_u_ = *K*_l_ = 4 × 10^5^ J m^−3^, the increase of DW velocity is depressed when *J* exceeds *J*_b_, 3.7 × 10^12^ A m^−2^. Nevertheless, with *K*_l_ = 2 × 10^5^ J m^−3^, the DW velocity keeps increasing when *J* is far beyond 4 × 10^12^ A m^−2^, indicating the depression of Walker breakdown under a higher current (the inset figure of Fig. 1(c)). And the reversal time of magnetic moments is about 0.01 ns.

**Fig. 2:**
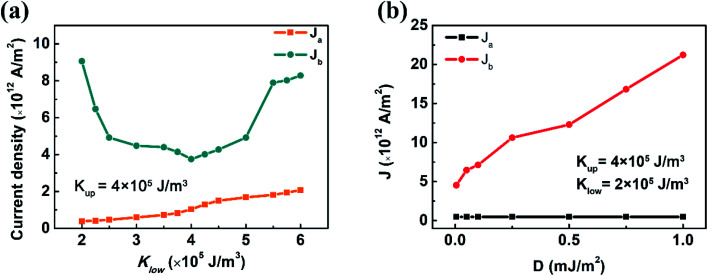
Critical current density *J*_a_ and *J*_b_ as a function of (a) the anisotropy constant of the lower layer *K*_l_ (*K*_u_ is fixed at 4 × 10^5^ J m^−3^) and (b) DMI constant *D*.

The variation of *J*_a_ and *J*_b_ with *K*_l_ (*K*_u_ is fixed at 4 × 10^5^ J m^−3^) is different (Fig. 2(a)). When *K*_l_ increases from 2 × 10^5^ J m^−3^ to 6 × 10^5^ J m^−3^, *J*_a_ increases monotonically from 5 × 10^11^ A m^−2^ to around 2 × 10^12^ A m^−2^. However, *J*_b_ changes with *K*_l_ non-monotonically: *J*_b_ is close to 1 × 10^13^ A m^−2^ when *K*_l_ is 2 × 10^5^ J m^−3^. It decreases with increasing *K*_l_ and reaches the minimum value when *K*_l_ = *K*_u_ and is enhanced again with further increase in *K*_l_. When *K*_l_ and *K*_u_ are fixed at 2 × 10^5^ J m^−3^ and 4 × 10^5^ J m^−3^, *J*_b_ can be further modified by manipulating *D* (Fig. 2(b)). When *D* increases from 0.05 mJ m^−2^ to 1 mJ m^−2^, *J*_a_ does not change, but *J*_b_ keeps increasing to 2 × 10^13^ A m^−2^, which is far beyond the upper limit of electrical current density for current electronic devices. It is noticed that this *D* is reasonable in experiments for some heavy metal/FM multilayers such as Pt/Co.^19,20^

**Fig. 3:**
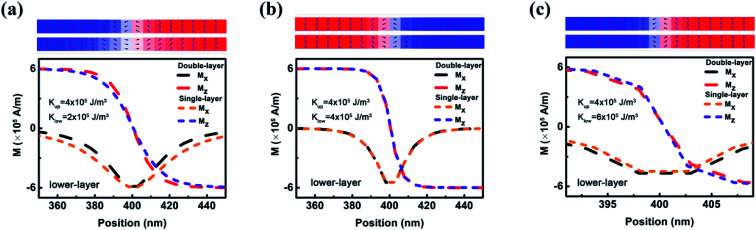
*x* and *z* components of magnetization near the centre of the lower DW in the double nanowire system with a fixed *K*_u_ (4 × 10^5^ J m^−3^) and different *K*_l_ ((a) *K*_l_ = 2 × 10^5^ J m^−3^, (b) *K*_l_ = 4 × 10^5^ J m^−3^, and (c) *K*_l_ = 6 × 10^5^ J m^−3^) and that in the single nanowire with the same *K*_l_. The upper panels show the configuration of double nanowire magnetic moments in the range of position coordinates.

In theory, when *β* is larger than *α*, the DW in a single FM nanowire is able to be driven if only the current density is higher than zero.^8,16^ However, the inter-wire magnetostatic coupling in the double FM nanowire system pins the DW motion and results in a nonzero *J*_a_.^16^ When *K*_l_ is different from *K*_u_, the inter-wire magnetostatic coupling may assist or hinder the motion of the lower DW, depending on the difference between *K*_l_ and *K*_u_. As indicated in Fig. 3, the structure of the lower DW in the double-nanowire system is modified due to the magnetostatic coupling from the upper DW. When *K*_l_ is 2 × 10^5^ J m^−3^, the lower DW in the double nanowire system is narrower than that in the single nanowire with the same parameters. This means that the magnetostatic coupling rotates the magnetic moments in the left part of the lower DW towards the +*z* axis. In the CIDWM, the DW is triggered to move to the right through the STT-driven rotation of the magnetic moments at the left of the DW towards the +*z* axis. Therefore, when *K*_l_ is 2 × 10^5^ J m^−3^, the magnetostatic coupling assists this rotation of magnetic moments, and the DW is able to move under a weaker current. When *K*_l_ is 6 × 10^5^ J m^−3^, higher than *K*_u_, the situation is the opposite: the magnetostatic coupling from the upper DW widens the lower DW and hinders the rotation of magnetic moments.

**Fig. 4:**
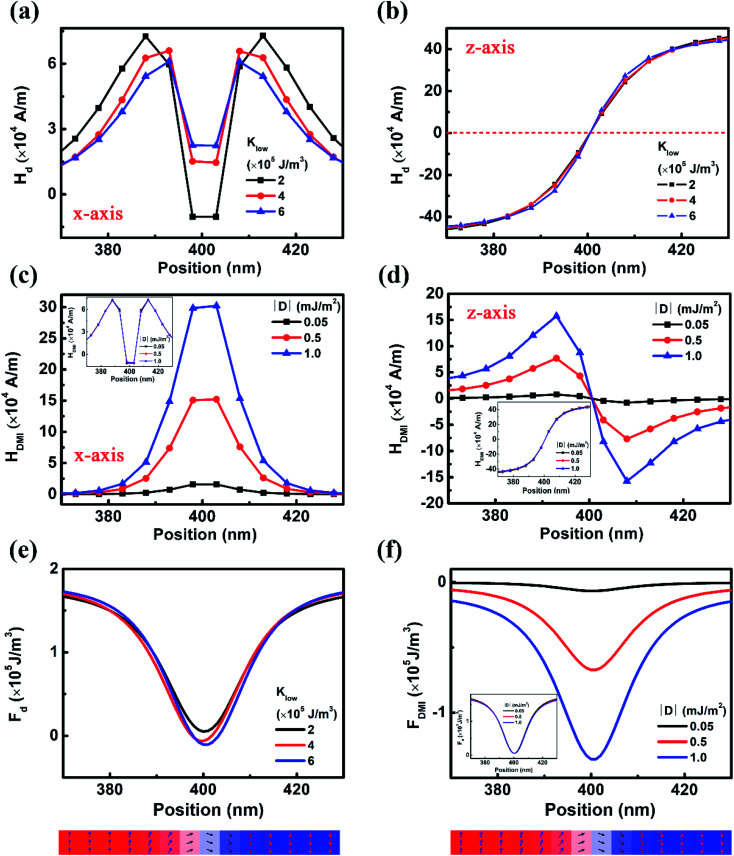
(a) *x* and (b) *z* components of the demagnetizing field in the DW region with different *K*_l_; (c) *x* and (d) *z* components of the DMI effective field in the DW region with different *D* (inset: (c) *x* and (d) *z* components of the demagnetizing field with different *D*); (e) density of demagnetizing energy in the DW region with different *K*_l_ and (f) energy density of DMI in the DW region with different *D* (inset: density of demagnetizing energy with different *D*). The lower panels show the configuration of magnetic moments in the range of position coordinates as shown in (a) to (f).

To understand the non-monotonic variation of *J*_b_, one needs to consider the variation of the magnetostatic coupling acting on the upper DW. This may be indirectly reflected from the demagnetizing field (*H*_d_) near the DW in the initial state. Because of the Néel-type DW structure, we only consider the *H*_d_ in the *xz* plane. The *x* and *z* components of *H*_d_ are extracted from the OOMMF. It is shown that changing *K*_l_ has a small effect on (*H*_d_)_*z*_, but changes (*H*_d_)_*x*_ considerably. When *K*_l_ increases from 2 × 10^5^ J m^−3^ to 6 × 10^5^ J m^−3^, the (*H*_d_)_*x*_ near the centre of the DW is enhanced, while that near the DW edge decreases. This indicates that when *K*_l_ is low or high, a strong (*H*_d_)_*x*_ assists the rotation of the magnetic moments either in the centre or at the edge of the DW. Since the DMI effective field is also in the *xz* plane, the changing of DMI also modifies the effective field for assisting/hindering the rotation of magnetic moments near the DW region. Both *x* and *z* components of the DMI effective field ((*H*_DMI_)_*x*_ and (*H*_DMI_)_*z*_) are enhanced with increasing *D* (Fig. 4(c) and (d)), but *H*_d_ does not change obviously (the inset figures in Fig. 4(c) and (d)). The increasing (*H*_DMI_)_*z*_ at the left edge of the DW assists the rotation of magnetic moments towards the +*z* direction, which makes it easy for the upper DW to move to the right and enhances *J*_b_.

The non-monotonic changing of *J*_b_ may also be understood from the aspect of free energy density. When *K*_l_ = 2 × 10^5^ J m^−3^, the demagnetizing energy density (*F*_d_) throughout the upper DW region is higher than that for *K*_l_ = 4 × 10^5^ J m^−3^. When *K*_l_ = 6 × 10^5^ J m^−3^, the *F*_d_ at the left side of the upper DW is also higher than that for *K*_l_ = 4 × 10^5^ J m^−3^ (Fig. 4(e)). Therefore, in both cases, the magnetic moments at the left side of the upper DW have a higher demagnetizing energy, which increases the probability for the rotation and the DW motion to the right. On the other hand, the energy density of DMI near the DW region increases with increasing *D* (Fig. 4(f)), yet the changing of DMI has little impact on the *F*_d_ (the inset figure of Fig. 4(f)). This also confirms that in addition to demagnetizing field, DMI also contributes to the enhancement of *J*_b_.

## Conclusion

In summary, in a coupled FM nanowire system, when the current is injected into the nanowire whose magnetic anisotropy constant is moderately lower than that of the other one, the DW can be triggered to move under a weaker current with a more stable magnetostatic coupling between the DWs in the two nanowires. This inter-layer magnetostatic coupling stabilizes the DW structure, depresses the Walker breakdown, and widens the range of current density for the DW motion. This paves a way to develop a novel racetrack memory device with low dissipation and high reading speed.

## Conflicts of interest

There are no conflicts to declare.

## Supplementary Material
